# Enhancement of Antioxidant Activity, Stability, and Structure of Heme-Peptides by L-Lysine

**DOI:** 10.3390/foods14020192

**Published:** 2025-01-09

**Authors:** Yinghui Zhang, Wei Cui, Hui Zhou, Lifang Zou, Zhaoming Wang, Kezhou Cai, Baocai Xu

**Affiliations:** 1 Key Laboratory for Animal Food Green Manufacturing and Resource Mining of Anhui Province, Hefei University of Technology, Hefei 230601, China; zhangyinghui218@163.com (Y.Z.); cuiwei5181@163.com (W.C.); zoulifang526@163.com (L.Z.); zhaomingwanghfut@163.com (Z.W.); baocaixu@163.com (B.X.); 2 School of Food and Biological Engineering, Hefei University of Technology, Hefei 230601, China; kzcai@hfut.edu.cn; 3 Engineering Research Center of Bio-Process, Ministry of Education, Hefei University of Technology, Hefei 230601, China

**Keywords:** by-products, heme-peptides, L-lysine, stability, structure

## Abstract

Porcine blood is rich in protein and has always been the focus of research. Heme-peptides prepared from porcine hemoglobin are susceptible to oxidative degeneration during preparation and storage, thus affecting their function and stability. This study evaluated the enhancement effects of L-lysine (Lys) on recovery rate, antioxidant activity, stability, and structure. The results indicated that adding 1% Lys during enzymatic hydrolysis significantly increased the recovery rate of ferrous heme and peptide content by 93.88% and 15.30% (*p* < 0.05), respectively, and maximally enhanced antioxidant activity by 37.85% (*p* < 0.05). The contents of iron, ferrous ion, and ferrous heme in the heme-peptides were significantly increased by 97.52%, 121. 97%, and 74.45% (*p* < 0.05), respectively. Additionally, Lys improved the resistance to pH, temperature, metal ions, pepsin, and trypsin. Meanwhile, the effects of Lys resulted in heme-peptides with a smaller particle size, higher zeta potentials, and a smoother micromorphology. Fourier-transform infrared spectroscopy and fluorescence spectroscopy analysis showed that Lys enhanced the conformational stability of the heme-peptides. Molecular docking further suggested that hydrogen bonding was the main driver of the connections between Lys and the heme-peptides. This study provides theoretical guidance for the efficient utilization of heme-peptides in the food industry.

## 1. Introduction

The blood of livestock and poultry is one of the main by-products of slaughter and processing and is rich in proteins and minerals [1]. Hemoglobin (Hb) is the most abundant protein in blood cells and is composed of globin and heme [2]. Globin is an excellent source of various bioactive peptides, and heme is a typical iron-containing porphyrin compound [3], which is highly effective in iron supplementation [4] and has anti-cancer [5], color enhancement [6], and other effects. However, heme is insoluble in water, limiting its wide application in the food industry [7]. Many studies have found that the heme-peptides produced by the enzymic hydrolysis of Hb, consisting of active peptides and heme, have many similar functional properties to heme [4,7,8]. Importantly, heme-peptides have better water solubility, fewer gastrointestinal side effects, and higher bioavailability compared to pure heme [9] and Hb [7]. However, heme-peptides are susceptible to factors such as pH, temperature, and metal ions, resulting in the oxidation of ferrous elements in heme to ferric iron during enzymatic hydrolysis and storage [10,11], which is not conducive to human digestion and absorption. Therefore, it is urgent to enhance the function and stability of heme-peptides.

Some studies have shown that nitric oxide (NO) [12] or carbon monoxide (CO) [13] can form a more stable structure with heme, which effectively prevents myoglobin from oxidizing to metmyoglobin [14], thereby improving color protection and stability. However, both CO and NO are toxic, limiting their application in the preparation and storage of heme-peptides [15,16]. Thus, a safe and effective approach needs to be developed.

The addition of amino acids during enzymatic hydrolysis is a feasible alternative route. L-lysine (Lys) is widely regarded as the “first essential amino acid” and demonstrates excellent DPPH scavenging activity, OH radical scavenging capacity, and strong iron-chelating activity [17,18,19]. Thus, we hypothesize that Lys has the potential to enhance the function and stability of heme-peptides during enzymatic hydrolysis and storage.

Therefore, our work aims to investigate the impact of Lys on enhancing the recovery rate and antioxidant activity of heme-peptides during preparation and study the effects of storage time, metal ions, temperature, pH, and simulated gastrointestinal digestion in vitro on the stability of heme-peptides. Furthermore, the effects of Lys on the structure of heme-peptides were analyzed using particle size, zeta potential, spectroscopy, and SEM. This study offers a theory for the preparation and usage of heme-peptides in the food industry.

## 2. Materials and Methods

### 2.1. Materials

Porcine blood was provided by Hefei Chunran Meat Food Co., Ltd. (Hefei, China). The quarantined fresh porcine blood was anticoagulated with sodium citrate (0.4%) and centrifuged at 5000 rpm and 4 °C. The deposited red blood cells (>99%) were cleaned twice with sterile saline and preserved at −20 °C overnight for reserve use. Lys (99%) and sodium citrate were obtained from Sigma (St. Louis, MO, USA), and alkaline protease (activity 200,000 U/g) was purchased from Beijing Solarbio Science & Technology Co., Ltd. (Beijing, China). All other chemical reagents were of analytical grade.

### 2.2. Sample Preparation

The method of producing heme-peptides through enzymatic hydrolysis was determined based on the method of He et al. [9]. The thawed red blood cells were blended with water at a ratio of 10:13 (*w*/*w*), and different concentrations of Lys (0, 0.05%, 0.1%, 0.5%, 1%, and 2%) were added, in turn. After reacting them for 1 h, the pH was set to 10, and the alkaline protease was added at a concentration of 3.14 g per 100 mL of the mixture. The mixture was then incubated for 5 h at 45 °C, then raised to 80 °C for 10 min to cease enzymatic activity. After being centrifuged at 5000 rpm for 15 min, the supernatant was then collected for the determination of ferrous heme recovery rate and peptide content.

### 2.3. Fractionation of Supernatant 

The supernatant obtained after enzymatic hydrolysis was placed in the funnel and further filtered through double-filter paper to collect the filtrate. The filtrate was further separated by an ultrafiltration cup (UFSC05001, Amicon stirred cell, Merck, Germany) and successively passed through membranes with pore sizes of 10 kDa and 3 kDa. The three components obtained by ultrafiltration (M1 < 3 kDa, 3 kDa < M2 < 10 kDa, and M3 > 10 kDa) were spray-dried by a spray-dryer (TENLIN-6000Y, spray-dryer, Yancheng, China). The content of ferrous heme and peptides was determined.

After ultrafiltration, the heme-peptides were marked as follows, based on their composition: Lys-HP (1% Lys) and HP (no Lys).

### 2.4. Recovery Rate and Composition

#### 2.4.1. Recovery Rate of Ferrous Heme

The recovery rate of ferrous heme was determined according to the procedure mentioned in the study [20], with certain changes. The sample was blended with pyridine–NaOH (33% pyridine:0.1 M NaOH = 1:1) in a ratio of 1:4 (*w*/*w*), and the absorbance was determined at 557 nm by a spectrophotometer (H1M, DARUI Co., Ltd., Guangzhou, China). Hemin served as a standard for quantifying the content of ferrous heme.

$$\mathrm{Recovery}\;\mathrm{rate}\;\mathrm{of}\;\mathrm{ferrous}\;\mathrm{heme}\left(\%\right)=\frac{M_{1}}{M_{0}}\;\times \;100$$

where M_1_ denotes the ferrous heme content in the supernatant of the enzymolysis solution after centrifugation (mg), and M_0_ denotes the mass of ferrous heme before enzymatic hydrolysis (mg).

#### 2.4.2. Peptide Content

The OPA reagent was made based on the method of Li et al. [21]. The sample was blended with the OPA solution in a ratio of 2:15 (*w*/*w*) and incubated for 2 min. The absorbance of the mixture was then measured at 340 nm on a spectrophotometer (H1M, DARUI Co., Ltd., Guangzhou, China). L-leucine was used as a standard substance to quantify the content of peptides.

#### 2.4.3. Ferrous Ion and Iron

The ferrous ion content was determined using a Ferrous Ion Content Assay Kit (Solarbio, Beijing, China), following the approach of Wang et al. [22]. The iron content was measured with an atomic absorption spectrophotometer (ICE-3000, Thermo Fisher Scientific, Waltham, MA, USA) at an inspection center (Anhui Zhongqing Inspection and Testing Co., Ltd., Hefei, China).

### 2.5. Determination of Antioxidant Activity

#### 2.5.1. DPPH Radical Scavenging Activity

The DPPH radical scavenging activity was assessed based on the methodology of previous studies, with slight changes [23,24]. The sample (1 mg/mL) was blended with 0.2 mM DPPH solution at a 1:1 ratio. The mixture was blended vigorously and reacted for 30 min. The absorbance was determined at 517 nm with a spectrophotometer (H1M, DARUI Co., Ltd., Guangzhou, China). The DPPH radical scavenging activity was determined as follows:

$$\mathrm{DPPH}\;\mathrm{radical}\;\mathrm{scavenging}\;\mathrm{activity}\left(\%\right)=\left(1\;-\;\frac{B_{1}\;-\;B_{2}}{B_{0}}\right)\;\times \;100$$

where B_1_ is the absorbance of the sample, B_2_ is the absorbance of the sample only (ethanol replacing the DPPH solution), and B_0_ is the absorbance of the control (deionized water replacing the sample).

#### 2.5.2. Reducing Power

The method of measuring the reducing power was the same as that used in a previous study [25], with some adjustments. The samples were mixed with 0.2 M phosphate buffer (pH 6.7) and 1% (*w*/*v*) potassium ferricyanide in a ratio of 1:1:1. The mixture was then reacted for 20 min at 50 °C. The reaction was terminated by the addition of trichloroacetic acid and centrifuged. The supernatant was blended with water and 0.1% (*w*/*v*) ferric chloride in a ratio of 5:5:2, then incubated for 10 min. Absorbance was determined with a spectrophotometer (H1M, DARUI Co., Ltd., Guangzhou, China) at 700 nm.

#### 2.5.3. Iron-Chelating Activity

The iron-chelating activity was identified based on the approach of a previous study [26], with some changes. The 2 mL sample was blended with 0.1 mL of 2 mM FeCl_2_ solution and 0.4 mL of 5 mM phenanthroline (deionized water served as the control). After incubating for 10 min, the absorbance of the mixture was determined at 562 nm using a spectrophotometer (H1M, DARUI Co., Ltd., Guangzhou, China). The iron-chelating activity was determined using the following formula:

$$\mathrm{Iron}-\mathrm{chelating}\;\mathrm{activity}\left(\%\right)=\left(\frac{S_{0}\;-\;S_{1}}{S_{0}}\right)\;\times \;100$$

where S_0_ and S_1_ represent the absorbance of the control and that of the sample, respectively.

#### 2.5.4. ABTS^·+^ Radical Scavenging Activity

The ABTS^·+^ radical scavenging activity was measured following the approach in a previous study [27], with some changes. The samples were mixed with ABTS^·+^ solution in a ratio of 1:2. After a reaction of 6 min, the absorbance of the mixture was measured at 734 nm with a spectrophotometer (H1M, DARUI Co., Ltd., Guangzhou, China). The ABTS^·+^ radical scavenging activity of the sample was determined as follows:

$${\mathrm{ABTS}}^{\cdot +}\;\mathrm{radical}\;\mathrm{scavenging}\;\mathrm{activity}\;\left(\%\right)=\frac{H_{0}\;-\;H_{1}}{H_{0}}\;\times \;100$$

where H_0_ and H_1_ represent the absorbance of the control and that of the sample, respectively.

### 2.6. Determination of Structural Properties

#### 2.6.1. Size and Zeta Potential of Heme-Peptides

The concentration of the sample was adjusted to 1 mg/mL. Then, the particle size and zeta potential of the sample were measured by a Malvern Zetasizer Nano-ZS instrument (C-W10X10XB22, Malvern Instruments Co., Ltd., Malvern, UK) at room temperature.

#### 2.6.2. Fourier-Transform Infrared Spectroscopy of Heme-Peptides

The functional groups and structural characterization of the sample were characterized using a Fourier-transform infrared (FT-IR) instrument (L160000A, FT-IR Spectrometer, PerkinElmer, Waltham, MA, USA). The dried KBr and the sample were mixed in an agate mortar at a 100:1 mass ratio. Subsequently, the mixture was ground into a powder under the infrared lamp and compressed into 1 mm thick particles at 10–15 MPa. The FT-IR scanning range was set to 4000–400 cm^−1^ with 128 scans.

#### 2.6.3. Intrinsic Fluorescence Spectrometry of Heme-Peptides

The intrinsic fluorescence spectra of the sample (1 mg/mL) were determined by a fluorescence spectrophotometer (F-4700, Fluorescence Spectrometer, Hitachi, Tokyo, Japan). The emission spectra ranged from 540 nm to 580 nm with an excitation wavelength of 280 nm, using an emission slit width of 5 nm.

#### 2.6.4. Scanning Electron Microscopy

The morphology and surface structure of the sample were investigated by a scanning electron microscope (SEM) (Regulus 8230, scanning electron microscopy, Hitachi, Tokyo, Japan). The spray-dried samples were attached to brass columns with conductive adhesive tape and subsequently plated with a coat of gold under a vacuum. The morphological characteristics were observed at an acceleration voltage of 20 KV.

#### 2.6.5. Molecular Docking Study

To further investigate the interactions, Hb was selected for the molecular docking study [28]. The crystal structure (Protein Data Bank [PDB]: 1QPW) was gained from the PDB (https://www.rcsb.org/structure/1QPW) (accessed on 10 May 2024). Before docking with Lys, water molecules were eliminated from the crystal by PyMol (1.8.6), and hydrogen atoms and charges were added using Autodock (4.2.6) [23]. The ligand was drawn as a secondary structure using Chem Draw 18.0. The 2D and 3D structures of the interaction between the heme-peptides and Lys were visualized using BIOVIA Discovery Studio 2021 Client (Dassault Systèmes, Paris, France). The interaction energies at the optimal docking positions were utilized to study the interaction between Lys and Hb.

### 2.7. Stability Characterization of Heme-Peptides

#### 2.7.1. Storage Stability

The sample was stored at room temperature (25 °C) in the dark, and the changes in stability were measured at 0, 7, 21, 60, and 150 days. The changes in ferrous heme content, peptide content, and ABTS^·+^ radical scavenging activity were assessed as previously described.

#### 2.7.2. Metal Ion Stability

The effects of metal ions on the stability of the sample were assessed according to a previous study [25], with a few changes. The 1 mg/mL sample was mixed with metal ion solutions (10 mM), including K^+^ (KCl), Na^+^ (NaCl), Zn^2+^ (ZnSO_4_), Mg^2+^ (MgCl_2_), and Cu^2+^ (CuSO_4_) to assess their effects. After incubating for 1 h, the mixture was analyzed for ferrous heme content, peptide content, and ABTS^·+^ radical scavenging activity.

#### 2.7.3. Thermal Stability

The method for measuring thermal stability was based on a previous study [29]. The 1 mg/mL sample was heated in test tubes at different temperatures (40, 60, 80, and 100 °C) for 1 h, followed by rapid cooling in ice water. The sample without incubation (25 °C) served as the control group. The thermal stability was determined by measuring ferrous heme content, peptide content, and ABTS^·+^ radical scavenging activity.

#### 2.7.4. pH Stability

The method for determining pH stability was based on previous research [30], with some adjustments. The sample (1 mg/mL) was adjusted to different pH values (2, 4, 6, 8, and 10) and then incubated for 1 h. The sample without pH adjustment (pH 7) was used as the control group. The pH stability was assessed by measuring the ferrous heme content, peptide content, and ABTS^·+^ radical scavenging activity.

### 2.8. Simulated Gastrointestinal Digestion In Vitro

#### 2.8.1. Gastric Digestion

The simulated gastrointestinal digestive stability was evaluated using pepsin–pancreatin hydrolysis in vitro, following the method of a previous study [31]. The sample (1 mg/mL) and 4.0% of pepsin (3000 U/g) were added to the simulated gastric electrolyte solution. The mixtures were incubated for 2 h to simulate gastric digestion. A portion of the mixture was heated to 85 °C for 15 min to stop the reaction and then cooled quickly for further analysis. The remaining portion was reserved for subsequent intestinal digestion.

#### 2.8.2. Intestinal Digestion

The pH was raised to 7.5 to terminate the gastric digestion. Pancreatin (250 U/g), 0.01 mM bile salt, and 0.3 mM CaCl_2_ were then added to create a simulated intestinal fluid. The mixture was incubated for 2 h to simulate intestinal digestion. The reaction was halted by heating to 85 °C for 15 min, followed by cooling to room temperature. The hydrolysates of simulated gastric and intestinal digestion were centrifuged, and the supernatant was used to measure the ferrous heme content, peptide content, and ABTS^·+^ radical scavenging activity.

### 2.9. Statistical Analysis

Each experiment was repeated in triplicate, with the results presented as the mean “±” standard deviation. Statistical significance was assessed using the IBM SPSS Statistics 24 software (IBM Inc., Evanston, IL, USA). Variance analysis was conducted via ANOVA, with a significance level set at *p* < 0.05. Correlation analysis was conducted using Origin 9.0. The peak and secondary structures were analyzed by PeakFit (V4.12).

## 3. Results and Discussion

### 3.1. Lys-Assisted Enzymolytic Preparation of Heme-Peptides

#### 3.1.1. Effects of Lys Concentration on the Preparation of Heme-Peptides

**Figure 1:**
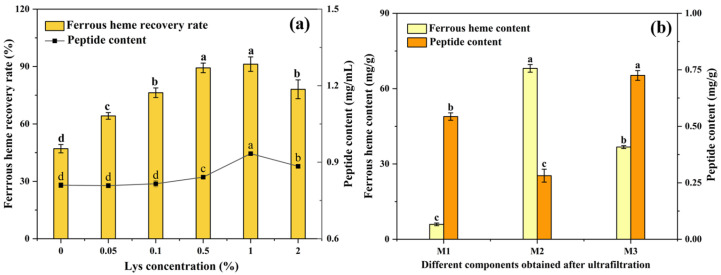
(**a**) Effects of Lys concentration on ferrous heme recovery rate and peptide content of the supernatant after enzymatic hydrolysis. (**b**) The ferrous heme content and peptide content of three components (M1 < 3 kDa, 3 kDa < M2 < 10 kDa, and M3 > 10 kDa) obtained by ultrafiltration. The results are presented as means ± SD (*n* = 3). Lowercase letters mean significant differences among different samples, with *p* < 0.05.

Both the ferrous heme recovery rate and the peptide content are important indexes to evaluate the extraction efficiency. Figure 1a shows the effects of Lys at different concentrations on the extraction efficiency. The ferrous heme recovery rate and peptide content significantly increased in the 0–1% concentration range, and, at a concentration of 1%, the ferrous heme recovery rate was 91.2 ± 3.78%, and the peptide content was 0.93 ± 0.002 mg/mL. They showed a downward trend when the concentration continued to increase to 2%. The increase could be attributed to the ability of Lys to chelate ferrous ions and scavenge radicals [18]. However, when the concentration was too high, the electrostatic interactions between Lys cations and negatively charged heme-peptides could disrupt intramolecular and intermolecular ionic linkages, thus weakening the protective effects [32]. Therefore, we used 1% Lys to assist the enzymatic hydrolysis of Hb.

#### 3.1.2. Ultrafiltration

The supernatant after Lys-assisted enzymatic hydrolysis was separated by 10 kDa and 3 kDa ultrafiltration membranes to obtain three components (M1 < 3 kDa, 3 kDa < M2 < 10 kDa, and M3 > 10 kDa). As shown in Figure 1b, the ferrous heme content of M2 was 68.07 ± 1.53 mg/g, which was significantly higher than that of M1 (5.96 ± 0.53 mg/g) and M3 (36.73 ± 0.58 mg/g) (*p* < 0.05). This might have been because the peptide of this component had the highest binding efficiency with ferrous heme, which was consistent with previous research [9]. Therefore, the M2 of the supernatant was further analyzed as heme-peptides.

#### 3.1.3. Composition Analysis

The composition of the HP and Lys-HP is evaluated in Table 1. The contents of iron, ferrous ion, and ferrous heme in HP were 2.42 ± 0.22 mg/g, 2.64 ± 0.90 μmol/L, and 39.02 ± 1.17 mg/g, respectively. The lower content could be attributed to its lower thermal stability. The HP was denatured and precipitated when the enzymatic activity was terminated by heating at 80 °C, which resulted in the enzymatic supernatant having a significantly lower content than that of Lys-HP. In addition, the high inlet and outlet temperatures during spray-drying also resulted in the oxidation of the heme-peptides. The content of iron, ferrous ion, and ferrous heme in Lys-HP significantly increased by 97.52%, 121.97%, and 74.45% (*p* < 0.05), respectively, compared to the HP. This suggested that Lys can improve the thermal stability of heme-peptides during the preparation process.

### 3.2. Antioxidant Activity

As depicted in Table 2, the DPPH scavenging capacity, reducing power, iron-chelating activity, and ABTS^·+^ radical scavenging activity of the HP were 14.32 ± 0.21%, 0.14 ± 0.01, 45.25 ± 1.31%, and 75.14 ± 0.62%, respectively. For Lys-HP, they were 19.74 ± 0.34%, 0.16 ± 0.01, 60.32 ± 0.80%, and 87.67 ± 1.75%, significantly higher than the HP (*p* < 0.05). Lys contained -COOH and -NR_2_ groups that could form tridentate chelates therefore it had a powerful ferrous ion-chelating ability, which could improve the antioxidant activity of heme-peptides and prevent the formation of free radicals [33]. Additionally, the amino group (-NH_2_) in Lys was easy to hydrogen bond with the amino acid residues (such as carboxylic groups) in heme-peptides, improving the structural stability, thereby enhancing the stability and functional performance [28].

### 3.3. Structural Properties

#### 3.3.1. Size and Zeta Potential

The particle size reflects the size of the sample, with smaller particles indicating greater system stability. As depicted in Table 3, the particle size of the heme-peptides changed from 229.14 nm (HP) to 179.38 nm (Lys-HP) after adding 1% Lys, a decrease of 27.74% (*p* < 0.05). This was in accordance with previous findings [30]. This decrease could be attributed to the following: (1) Lys may have interacted with the amino acid side chain of the heme-peptides to form partially unfolded peptide intermediates, thus effectively inhibiting the aggregation of said heme-peptides; and (2) the binding between the ionizable groups of Lys (α-COOH and α-NH_2_) and peptides may have improved the energy barrier of peptide aggregation, thus preventing it and decreasing the particle size [30,33].

The zeta potential is an essential physicochemical parameter reflecting the surface charge state of the particles in a dispersed system, with higher zeta potential values corresponding to increased stability. The zeta potential of Lys-HP was −39.65 mV, significantly higher than that of the HP (−25.38 mV), suggesting that the binding of Lys to heme-peptides improved electrostatic repulsion among the peptides [30]. This enhancement contributed to the stability of the heme-peptides.

#### 3.3.2. FT-IR Spectroscopy

**Figure 2:**
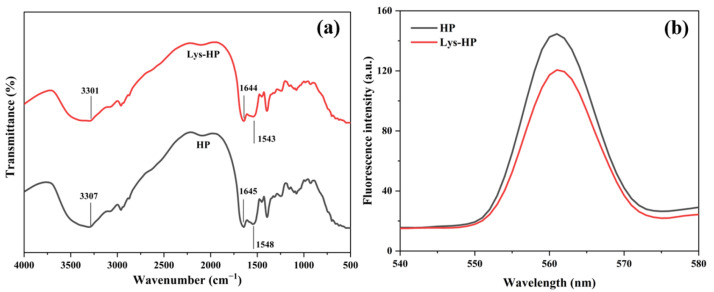
(**a**) FTIR spectra and (**b**) intrinsic fluorescence spectrometry of HP and Lys-HP.

FT-IR spectroscopy was conducted to study the functional groups and structural characterization of the HP and Lys-HP [34]. As shown in Figure 2a, the heme-peptides had two distinctive characteristic absorption bands: the amide I band at 1700–1600 cm^−1^ (C=O stretching vibration), and the amide II band at 1550–1500 cm^−1^ (N-H deformation). Lys led to weak red shifts in the peak absorptions, indicating that C=O and N-H had changed slightly. Previous studies indicated that iron ions could bind to peptides through carboxyl oxygen and amino nitrogen atoms [35]. Thus, the alterations in the characteristic peaks within the amide I and II regions could be attributed to more iron-binding sites, as evidenced by the enhanced iron-chelating activity of Lys-HP (Table 2). Furthermore, the peak frequency of the heme-peptides changed from 3307 cm^−1^ (HP) to 3301 cm^−1^ (Lys-HP) after adding Lys, suggesting that Lys had a significant influence on the O-H vibration, with a lower peak frequency indicating stronger interactions [36]. Xu et al. [37] observed that the intensity of the stretching band at 3500–3300 cm^−1^ decreased, showing that the number of hydrogen bonds and interactions within the macromolecular chains increased due to the physicochemical cross-linking process, which was consistent with our findings.

The influence of Lys on the secondary structure of the heme-peptides was determined in the amide I band (1600–1700 cm^−1^) based on the FT-IR spectrum. The secondary structure content of the HP and Lys-HP is shown in Table 3. The α-helix and β-sheet were increased in Lys-HP compared to HP, and the formation of β-sheets was mainly related to the interaction of intermolecular hydrogen bonds. Lys significantly improved the conformational stability of the heme-peptides by forming hydrogen bonds with the carbonyl of their amino acid side chain. Peptide chains with α-helical structures showed right-handed, coiled helices, and those with β-sheets structures were fully extended into a zigzag configuration, indicating a compact protein structure [38]. Thus, Lys promoted the conversion of disordered structures (random coil and β-turn) to ordered structures (β-sheet and α-helix), reducing structural randomness and enhancing the structural stability of the heme-peptides.

#### 3.3.3. Intrinsic Fluorescence Spectrometry

Intrinsic fluorescence spectrometry was conducted to further explore the interaction of the heme-peptides with Lys. Heme-peptides contain residues, which contribute to their intrinsic fluorescence and are highly sensitive to micro-environmental changes upon ligand binding [39]. By investigating the changes in fluorescence intensity, we could study the internal structural modifications of HP and Lys-HP.

As shown in Figure 2b, the fluorescence peak λmax of Lys-HP was 120.62, significantly lower than HP (144.63) (*p* < 0.05), possibly because Lys interacted with the amino acid side chain of the heme-peptides to form partially unfolded peptide intermediates, which efficiently suppressed the aggregation of the heme-peptides and improved their stability. Therefore, the residues were exposed to a polar microenvironment, which reduced the fluorescence intensity. This result was similar to previous findings [40].

#### 3.3.4. Micromorphology

**Figure 3:**
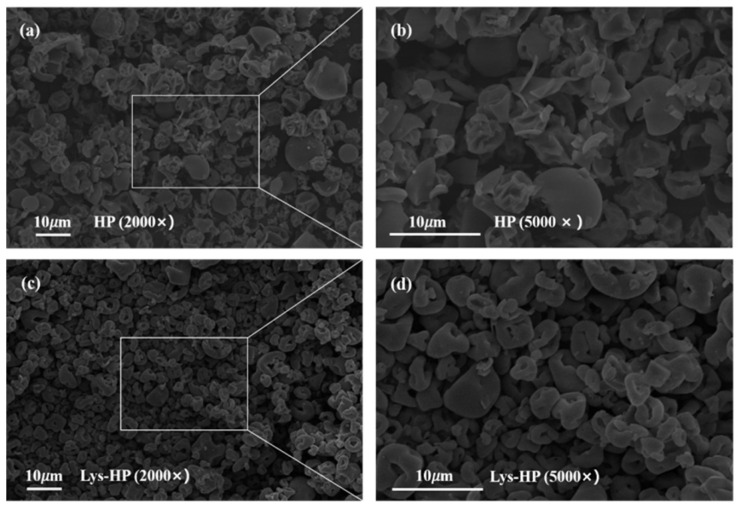
Scanning electron microstructure at 2000× and 5000× magnification of HP and Lys-HP, with (**b**) and (**d**) being magnifications of (**a**) and (**c**), respectively.

The micromorphology of HP and Lys-HP observed with scanning electron microscopy is shown in Figure 3. The spray-dried samples exhibited spherical shapes and a range of sizes, which are typical characteristics of particles produced through the spray-drying process. As can be noticed (Figure 3a,b), the particles of HP were semi-spherical and wrinkled, with noticeable dents on the surface. The wrinkles were caused by the spray-drying process, which generated steam pressure on the internal structure. This pressure led to rapid shrinkage as a result of moisture loss [41]. In contrast, compared to HP, the particles of Lys-HP exhibited a smooth surface and a spherical shape, with no wrinkles (Figure 3c,d).

Lys has functional groups -COOH and -NH_2_, facilitating hydrogen bonding with water molecules and reducing drip loss during spray-drying [42]. This finding indicates that adding Lys during preparation has a protective effect on heme-peptides.

#### 3.3.5. Molecular Docking Simulation Analysis

Molecular docking technology is crucial in studying the interaction between proteins and small molecules. It provides a more intuitive way to observe the structural changes in protein molecules, as well as the types of forces and binding conditions between proteins and small molecules [10,28].

**Figure 4:**
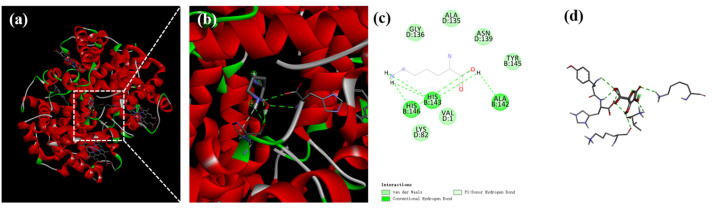
Binding interactions of Lys with Hb. Optimum docking conformations and interaction modes of (**a**–**d**) Lys with Hb, with (**b**) being a magnification of (**a**).

The molecular docking results are illustrated in Figure 4. The binding sites of Hb with Lys were located in the groove of Hb, acting as the active center. There was sufficient space within the hydrophobic cavity of Hb to accommodate Lys (Figure 4a,b). Lys interacted via hydrogen bonds with the amino acid residues of Hb, such as HIS-146, HIS-143, and ALA-142, as well as via van der Waals forces with GLY-136, ALA-135, ASN-139, TYR-145, LYS-82, and VAL-1 (Figure 4c,d). Lys and Hb formed more stable complexes through these various forces. The results of molecular docking were confirmed with the pattern results of Fourier-transform infrared spectroscopy, confirming that hydrogen bonds played a critical role in the interaction between Lys and Hb.

### 3.4. Stability Characterization Analysis

#### 3.4.1. Effects of Storage Time on Heme-Peptides

The storage stability of heme-peptides was investigated to ensure their validity and biological activity in the food industry. As shown in Figure S1a,b, the ferrous heme content, peptide content, and the ABTS^·+^ radical scavenging activity of both groups reduced significantly with the extension of storage time. This decreasing trend was consistent with previous findings [30]. This might have been due to the environmental factors, such as temperature, humidity, light, and oxygen concentration, which accelerated the oxidation and degradation of the heme-peptides during storage [43]. Interestingly, these indexes of Lys-HP were significantly higher than for HP after 150 days of storage, measuring 39.80 ± 1.15 mg/g, 0.16 ± 0.01 mg/g, and 68.69 ± 1.67 % (*p* < 0.05), respectively. These results indicated that Lys exhibited significant effects in terms of inhibiting the oxidation of the heme-peptides during storage.

#### 3.4.2. Effects of Metal Ions on Heme-Peptides

Some metal ions can be ligand-bound to heme-peptides, thus affecting their structure and function. As shown in Figure S1c,d, the heme-peptides exhibited different activities in various metal ion buffers. In the presence of K^+^, Na^+^, and Mg^2+^, the ferrous heme content, peptide content, and the ABTS^·+^ radical scavenging activity of both groups remained relatively constant, indicating that K^+^, Na^+^, and Mg^2+^ had minimal impact on the stability of the heme-peptides, which was similar to previous findings [25]. Transition metal ions, such as Cu^2+^ and Zn^2+^, reduced the peptide content and ABTS^·+^ radical scavenging activity of both groups due to their ability to catalyze the generation of reactive oxygen species (ROS) [44]. Zn^2+^ could chelate with protoporphyrin to form zinc protoporphyrin [45], thus reducing the ferrous content of both groups. In the presence of Cu^2+^, the ferrous heme content of both groups increased significantly. This might have been because electron transfer occurred after adding Cu^2+^, and the electrons were then transferred to the site bound to the heme-peptides, increasing the ferrous heme content. Interestingly, the ferrous heme content, peptide content, and the ABTS^·+^ radical scavenging activity of Lys-HP were significantly higher than those of HP. This could be attributed to the fact that Lys significantly improved the conformational stability of the heme-peptides by forming hydrogen bonds with their amino acid side chain, thereby increasing the resistance of the heme-peptides to metal interference.

#### 3.4.3. Effects of Temperature on Heme-Peptides

Thermal stability is a crucial prerequisite for using heme-peptides as functional food ingredients. As shown in Figure S1e,f, the ferrous heme content, peptide content, and the ABTS^·+^ radical scavenging activity of both groups remained relatively stable after incubation at 40 °C for 1 h. However, exposure to higher temperatures (60, 80, and 100 °C for 1 h) significantly reduced these indexes in both groups. This striking loss could be attributed to the degradation and aggregation of heme-peptides induced by high temperatures [25,46], consistent with the findings in Table 1. It was worth noting that, after incubation at 100 °C for 1 h, the ferrous heme content, peptide content, and the ABTS^·+^ radical scavenging activity of Lys-HP were 60.78 ± 0.55 mg/g, 0.21 ± 0.01 mg/g, and 70.29 ± 0.30 % (*p* < 0.05), respectively, representing a significant increase compared to HP. These results showed that adding Lys can enhance the thermal stability of heme-peptides, and this finding was consistent with the findings of Section 3.1.3.

#### 3.4.4. Effects of pH on Heme-Peptides

The pH has a substantial effect on both the structural stability and solubility of heme-peptides, so studying their stability under various pH conditions is crucial for determining the optimal conditions for their storage and use. As shown in Figure S1g,h, for alkaline conditions (pH 8 and 10), the ferrous heme content, peptide content, and ABTS^·+^ radical scavenging activity of HP and Lys-HP were not significantly different from those of the control group. The results indicated that it was beneficial to maintain the stability of the heme-peptides under alkaline conditions. In contrast, these properties in HP and Lys-HP were significantly reduced at pH levels of 2, 4, and 6 and were the lowest at pH 6. These reductions could be because heme is insoluble in acidic and neutral environments [47]. Furthermore, pH 6 is close to the isoelectric point of heme-peptides (5.8) [47,48], leading to heme-peptide precipitation. Interestingly, at pH 6, the ferrous heme content, peptide content, and ABTS^·+^ radical scavenging activity of Lys-HP were approximately increased by 122%, 134%, and 214% (*p* < 0.05), respectively, compared to HP, indicating that Lys can enhance the pH stability of heme-peptides.

### 3.5. Pepsin–Pancreatin Simulated Digestion In Vitro

**Figure 5:**
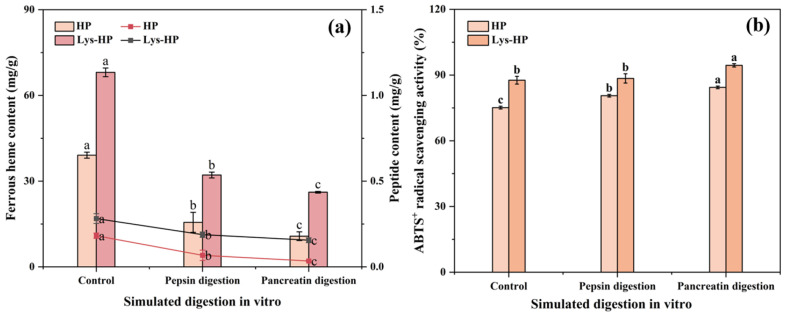
The effects of simulated pepsin–pancreatin digestion in vitro on (**a**) the ferrous heme content, the peptide content, and (**b**) the ABTS^·+^ radical scavenging activity of heme-peptides. Lower case letters indicate significant differences in content and activity of the same sample during simulated gastrointestinal digestion. (*p* < 0.05).

The study of the biological activity of heme-peptides after gastrointestinal digestion is of great significance for evaluating their functional stability. As shown in Figure 5, the ferrous heme content and peptide content of the heme-peptides significantly decreased during the simulated digestion. This reduction might be attributed to their further hydrolysis into shorter peptides and amino acids [49]. Additionally, the decline could be attributed to the strongly acidic condition (pH 2), which aligned with the results of Section 3.4.4. The ABTS^·+^ radical scavenging activity of both groups significantly enhanced after the simulated digestion, consistent with previous findings [25]. This may be because pepsin and pancreatin can further cleave the hydrolysate, exposing amino residue side chain groups which may efficiently trap ABTS radicals [42]. Notably, the digestive products of Lys-HP had better ferrous heme content, peptide content, and ABTS^·+^ radical scavenging activity than HP.

## 4. Conclusions

This study evaluated the enhancement effects of Lys on the recovery rate, antioxidant activity, stability, and structure of heme-peptides. The results indicated that the addition of 1% Lys led to the highest recovery rate of heme-peptides. The antioxidant activity was maximally increased by 37.85% and the contents of iron, ferrous ion, and ferrous heme in heme-peptides were increased by 97.52%, 121.97%, and 74.45% (*p* < 0.05), respectively. Additionally, Lys improved the tolerance to pH, temperature, and metal ions. Meanwhile, the enhancement effects of Lys resulted in heme-peptides with a smaller particle size, higher zeta potentials, and a smoother micromorphology. Fourier-transform infrared spectroscopy and fluorescence spectroscopy analyses showed that Lys enhanced the conformational stability of the heme-peptides by forming hydrogen bonds with their amino acid side chains, promoting the conversion of the disordered structure to an ordered structure. Molecular docking further suggested that hydrogen bonding was the main driver of the connections between Lys and the heme-peptides. Therefore, this study showed that the addition of Lys during the enzymatic hydrolysis of Hb can improve the function and stability of heme-peptides. The findings provide new insights into the production of heme-peptides with higher function and stability from livestock and poultry blood, which is important for the development of functional foods.

## Figures and Tables

**Table 1:** Composition of heme-peptides (dry basis).

Composition	Iron (mg/g)	Ferrous Ion (μmol/L)	Ferrous Heme (mg/g)
HP	2.42 ± 0.22 ^b^	2.64 ± 0.90 ^b^	39.02 ± 1.17 ^b^
Lys-HP	4.78 ± 0.20 ^a^	5.86 ± 1.35 ^a^	68.07 ± 1.53 ^a^

Different lowercase letters indicate significant differences (*p* < 0.05) between the HP and Lys-HP.

**Table 2:** Antioxidant activity in vitro of heme-peptides.

Sample	DPPH Scavenging Capacity (%)	Reducing Power (Abs)	Iron-Chelating (%)	ABTS^·+^ Radical Scavenging Activity (%)
HP	14.32 ± 0.21 ^b^	0.14 ± 0.01 ^b^	45.25 ± 1.31 ^b^	75.14 ± 0.62 ^b^
Lys-HP	19.74 ± 0.34 ^a^	0.16 ± 0.01 ^a^	60.32 ± 0.80 ^a^	87.67 ± 1.75 ^a^

Different lowercase letters indicate significant differences (*p* < 0.05) between the HP and Lys-HP.

**Table 3:** Size, zeta potential, and secondary structures of the heme-peptides.

Sample	Size (nm)	Zeta Potential (mV)	Secondary Structures (%)			
			β-Sheet	α-Helix	Random Coil	β-Turn
HP	229.14 ± 2.31 ^a^	−25.38 ± 1.21 ^b^	7.94	4.02	53.57	34.47
Lys-HP	179.38 ± 4.18 ^b^	−39.65 ± 1.31 ^a^	56.92	21.46	7.15	14.47

Different lowercase letters indicate significant differences (*p* < 0.05) between the HP and Lys-HP.

## Data Availability

The original contributions presented in the study are included in the article/Supplementary Materials, further inquiries can be directed to the corresponding author.

## Supplementary Materials

The following supporting information can be downloaded at https://www.mdpi.com/article/10.3390/foods14020192/s1: Figure S1. The effects of (a, b) time; (c, d) metal ions; (e, f) temperature; and (g, h) pH on the ferrous heme content, peptide content, and the ABTS^·+^ radical scavenging activity of heme-peptides.

## References

[1] Cheng C., Chen L., Zhang D., Yu J., Zhu M., Li C., Zheng X., Blecker C., Li S. (2024). Value-added utilization of hemoglobin and its hydrolysis products from livestock and poultry blood processing by-products: A review. Trends Food Sci. Technol..

[2] Liu L., Guo J., Wang Z., Duan X., Zhang X., Yu Y., Su K., Lu Y., Wu T. (2023). Effect of interaction between catechin and glycated porcine hemoglobin on its antioxidant functional and structural properties. LWT Food Sci. Technol..

[3] Wang B., Cheng F., Gao S., Ge W., Zhang M. (2017). Double enzymatic hydrolysis preparation of heme from goose blood and microencapsulation to promote its stability and absorption. Food Chem..

[4] Pasricha S.-R., Tye-Din J., Muckenthaler M.U., Swinkels D.W. (2021). Iron deficiency. Lancet.

[5] Park S.-H., Lee Y., Jeon H., Park J., Kim J., Kang M., Namkung W. (2024). Anticancer Effect of Hemin through ANO1 Inhibition in Human Prostate Cancer Cells. Int. J. Mol. Sci..

[6] Tian T., Wu X., Wu P., Lu X., Wang Q., Lin Y., Liu C., Zhou J., Yu Y., Lu H. (2024). High-level expression of leghemoglobin in *Kluyveromyces marxianus* by remodeling the heme metabolism pathway. Front. Bioeng. Biotechnol..

[7] Berraquero-García C., Almécija M.C., Guadix E.M., Pérez-Gálvez R. (2022). Valorisation of blood protein from livestock to produce haem iron-fortified hydrolysates with antioxidant activity. Int. J. Food Sci. Technol..

[8] Chang C.Y., Wu K.C., Chiang S.H. (2007). Antioxidant properties and protein compositions of porcine haemoglobin hydrolysates. Food Chem..

[9] He L., Yang F., Liang Y., Zhang M., Liu X., Zhao S., Jin G. (2020). Process optimisation of haemoglobin hydrolysis by complex proteases to produce haem-enriched peptides and its iron uptake property evaluation by Caco-2 cell model. Int. J. Food Sci. Technol..

[10] Hu X., Wu D., Tang L., Zhang J., Zeng Z., Geng F., Li H. (2022). Binding mechanism and antioxidant activity of piperine to hemoglobin. Food Chem..

[11] Tansukkasem S., Kaewpathomsri P., Jonjaroen V., Payongsri P., Lertsiri S., Niamsiri N. (2023). Production and Characterization of Heme Iron Polypeptide from the Blood of Skipjack Tuna (*Katsuwonus pelamis*) Using Enzymatic Hydrolysis for Food Supplement Application. Foods.

[12] Song X., Cornforth D., Whittier D., Luo X. (2015). Nitrite spray treatment to promote red color stability of vacuum packaged beef. Meat Sci..

[13] Zhou C., Ye H., Nishiumi T., Qin H., Chen C. (2014). l-Histidine enhances stability of hemoglobin concentrates by coordinating with free iron. Food Res. Int..

[14] Xu P., Zhu X., Tan S., Qin H., Zhou C. (2016). The role of monoxide hemoglobin in color improvement of chicken sausage. Food Sci. Biotechnol..

[15] Djenane D., Roncales P. (2018). Carbon Monoxide in Meat and Fish Packaging: Advantages and Limits. Foods.

[16] Karwowska M., Kononiuk A. (2020). Nitrates/Nitrites in Food-Risk for Nitrosative Stress and Benefits. Antioxidants.

[17] Guo X., Xu S., Jiang P., Fu C., Wang J., Meng X. (2024). L-lysine enhances pork color through antioxidant activity and myoglobin conformational changes. Food Res. Int..

[18] Ning C., Li L., Fang H., Ma F., Tang Y., Zhou C. (2019). l-Lysine/l-arginine/l-cysteine synergistically improves the color of cured sausage with NaNO_2_ by hindering myoglobin oxidation and promoting nitrosylmyoglobin formation. Food Chem..

[19] Zhu X., Li L., Li S., Ning C., Zhou C. (2019). l–Arginine/l–lysine improves emulsion stability of chicken sausage by increasing electrostatic repulsion of emulsion droplet and decreasing the interfacial tension of soybean oil-water. Food Hydrocoll..

[20] Barr I., Guo F. (2015). Pyridine Hemochromagen Assay for Determining the Concentration of Heme in Purified Protein Solutions. Bio Protoc..

[21] Li P., Xu F., Zhou H., Gao Y., Zhu H., Nie W., Wang Z., Wang Y., Deng J., Zhou K. (2022). Evolution of antioxidant peptides and their proteomic homology during processing of Jinhua ham. LWT Food Sci. Technol..

[22] Wang Y., Tan L., Zhang W., Yang Y., Li C., Li H., Cai K., Hu Y., Luo Z., Liu M. (2023). Injectable Dynamic Hydrogel with Responsive Mechanical Reinforcing Ability Reverses Intervertebral Disc Degeneration by Suppressing Ferroptosis and Restoring Matrix Homeostasis. Adv. Funct. Mater..

[23] Chen G., Wang S., Feng B., Jiang B., Miao M. (2019). Interaction between soybean protein and tea polyphenols under high pressure. Food Chem..

[24] Zhu H., Yi X., Jia S.S., Liu C.Y., Han Z.W., Han B.X., Jiang G.C., Ding Z.F., Wang R.L., Lv G.P. (2023). Optimization of Three Extraction Methods and Their Effect on the Structure and Antioxidant Activity of Polysaccharides in Dendrobium huoshanense. Molecules.

[25] Zheng Z., Si D., Ahmad B., Li Z., Zhang R. (2018). A novel antioxidative peptide derived from chicken blood corpuscle hydrolysate. Food Res. Int..

[26] Ding X., Li H., Xu M., Li X., Li M. (2024). Peptide composition analysis, structural characterization, and prediction of iron binding modes of small molecular weight peptides from mung bean. Food Res. Int..

[27] Zhang Q., Tong X., Qi B., Wang Z., Li Y., Sui X., Jiang L. (2018). Changes in antioxidant activity of Alcalase-hydrolyzed soybean hydrolysate under simulated gastrointestinal digestion and transepithelial transport. J. Funct. Foods.

[28] Zhu B., Yang J., Yu J., Dou J., Ning Y., Qi B., Li Y. (2024). Effects of l-arginine/l-lysine modifications on the protein structure, binding interactions, and functional properties of soy protein hydrolysate. Food Hydrocoll..

[29] Zhang S.Y., Zhao G.X., Suo S.K., Wang Y.M., Chi C.F., Wang B. (2021). Purification, Identification, Activity Evaluation, and Stability of Antioxidant Peptides from Alcalase Hydrolysate of Antarctic Krill (*Euphausia superba*) Proteins. Mar. Drugs.

[30] Zhu D., Cheng S., Du M. (2024). Oxidation-resistant nanoliposomes loaded with osteogenic peptides: Characteristics, stability and bioaccessibility. Food Res. Int..

[31] Jiang H., Xu Y., Chen G., Liu T., Yang Y., Mao X. (2024). Digestive properties and peptide profiles exhibited significant differences between skim camel milk and bovine milk powder after static in vitro simulated infant gastrointestinal digestion. Food Res. Int..

[32] Guo X.Y., Peng Z.Q., Zhang Y.W., Liu B., Cui Y.Q. (2015). The solubility and conformational characteristics of porcine myosin as affected by the presence of L-lysine and L-histidine. Food Chem..

[33] Guo X., Wu J., Meng X., Zhang Y., Peng Z. (2022). Oxidative characteristics and gel properties of porcine myofibrillar proteins affected by l-lysine and l-histidine in a dose-dependent manner at a low and high salt concentration. Int. J. Food Sci. Technol..

[34] Meng C., Li S., Zhang D., Liu H., Sun B. (2024). Conjugated molecularly imprinted polymers based on covalent organic frameworks: Fluorescent sensing platform for specific capture of urea and elimination of ethyl carbamate. Spectrochim. Acta Part A.

[35] Sun N., Cui P., Jin Z., Wu H., Wang Y., Lin S. (2017). Contributions of molecular size, charge distribution, and specific amino acids to the iron-binding capacity of sea cucumber (*Stichopus japonicus*) ovum hydrolysates. Food Chem..

[36] Su X., Cui W., Zhang Z., Zhang J., Zhou H., Zhou K., Xu Y., Wang Z., Xu B. (2023). Effects of L-lysine and L-arginine on the structure and gel properties of konjac glucomannan. Food Hydrocoll..

[37] Xu N., Yang H., Wei R., Pan S., Huang S., Xiao X., Wen H., Xue W. (2019). Fabrication of Konjac glucomannan-based composite hydrogel crosslinked by calcium hydroxide for promising lacrimal plugging purpose. Int. J. Biol. Macromol..

[38] Sun J., Liu T., Zhang F., Huang Y., Zhang Y., Xu B. (2022). Tea polyphenols on emulsifying and antioxidant properties of egg white protein at acidic and neutral pH conditions. LWT Food Sci. Technol..

[39] Dong J., Zhou Y., Lu Y., Lv Y., Chi Y., He Q. (2019). Effect of Tea Polyphenols on the Oxidation and Color Stability of Porcine Hemoglobin. J. Food Sci..

[40] Li S., Li L., Zhu X., Ning C., Cai K., Zhou C. (2019). Conformational and charge changes induced by l-Arginine and l-lysine increase the solubility of chicken myosin. Food Hydrocoll..

[41] Insang S., Kijpatanasilp I., Jafari S., Assatarakul K. (2022). Ultrasound-assisted extraction of functional compound from mulberry (*Morus alba* L.) leaf using response surface methodology and effect of microencapsulation by spray drying on quality of optimized extract. Ultrason. Sonochemistry.

[42] Wachirasiri K., Wanlapa S., Uttapap D., Rungsardthong V. (2016). Use of amino acids as a phosphate alternative and their effects on quality of frozen white shrimps (*Penaeus vanamei*). LWT Food Sci. Technol..

[43] Zhang G., Fang S., Regenstein J.M., Wang F. (2022). Preparation, characterization and stability of nanoliposomes loaded with peptides from defatted walnut (*Juglans regia* L.) meal. J. Food Sci. Technol..

[44] Atrian-Blasco E., Conte-Daban A., Hureau C. (2017). Mutual interference of Cu and Zn ions in Alzheimer’s disease: Perspectives at the molecular level. Dalton Trans..

[45] Ghadiri Khozroughi A., Kroh L.W., Schluter O., Rawel H. (2018). Assessment of the bacterial impact on the post-mortem formation of zinc protoporphyrin IX in pork meat. Food Chem..

[46] Vraneš M., Panić J., Tot A., Papović S., Gadžurić S., Podlipnik Č., Bešter-Rogač M. (2021). From amino acids to dipeptide: The changes in thermal stability and hydration properties of β-alanine, L-histidine and L-carnosine. J. Mol. Liq..

[47] Liu T.X., Wang J., Zhao M.M. (2010). In vitro haem solubility of red cell fraction of porcine blood under various treatments. Int. J. Food Sci. Technol..

[48] In M.-J., Kim D.C., Chae H.J., Oh N.-S. (2003). Effects of degree of hydrolysis and pH on the solubility of heme-iron enriched peptide in hemoglobin hydrolysate. Biosci. Biotechnol. Biochem..

[49] You L., Zhao M., Regenstein J.M., Ren J. (2010). Changes in the antioxidant activity of loach (*Misgurnus anguillicaudatus*) protein hydrolysates during a simulated gastrointestinal digestion. Food Chem..

