# Supplementary material for: Enhancement of Antioxidant Activity, Stability, and Structure of Heme-Peptides by L-Lysine
Source: Foods. 2025 Jan 9;14(2):192. doi: 10.3390/foods14020192 (PMC11764818; doi:10.3390/foods14020192)
Supplement: Supplementary file 1 [file foods-14-00192-s001.zip › foods-3366916-supplementary.pdf]

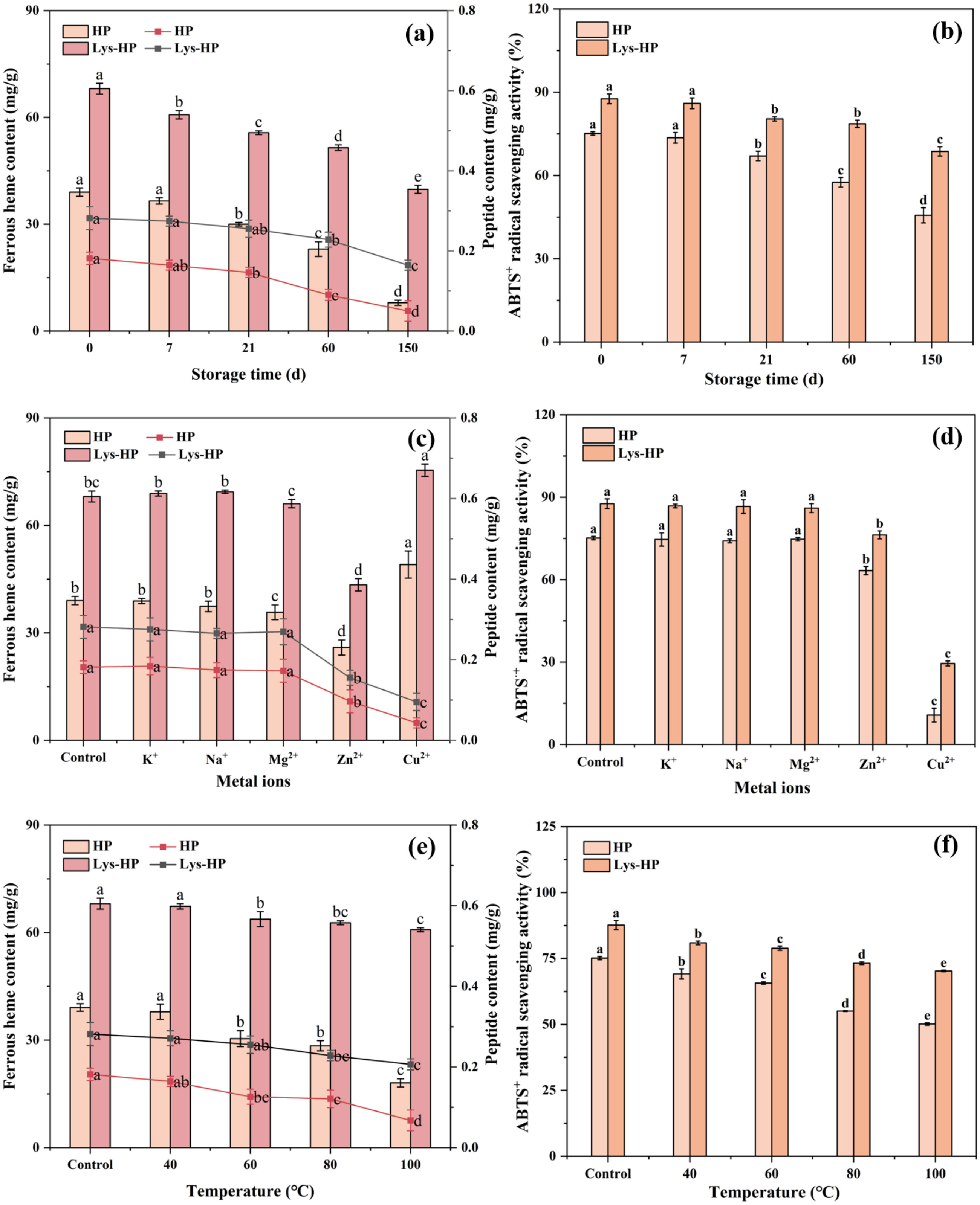
(a)
HP
Lys-HP
Ferrous heme content (mg/g)
Peptide content (mg/g)
Storage time (d)
0
7
21
60
150
(b)
ABTS+ radical scavenging activity (%)
Storage time (d)
(c)
Ferrous heme content (mg/g)
Peptide content (mg/g)
Metal ions
Control
K+
Na+
Mg2+
Zn2+
Cu2+
(d)
ABTS·+ radical scavenging activity (%)
Metal ions
(e)
Ferrous heme content (mg/g)
Peptide content (mg/g)
Temperature (°C)
Control
40
60
80
100
(f)
ABTS+ radical scavenging activity (%)
Temperature (°C)

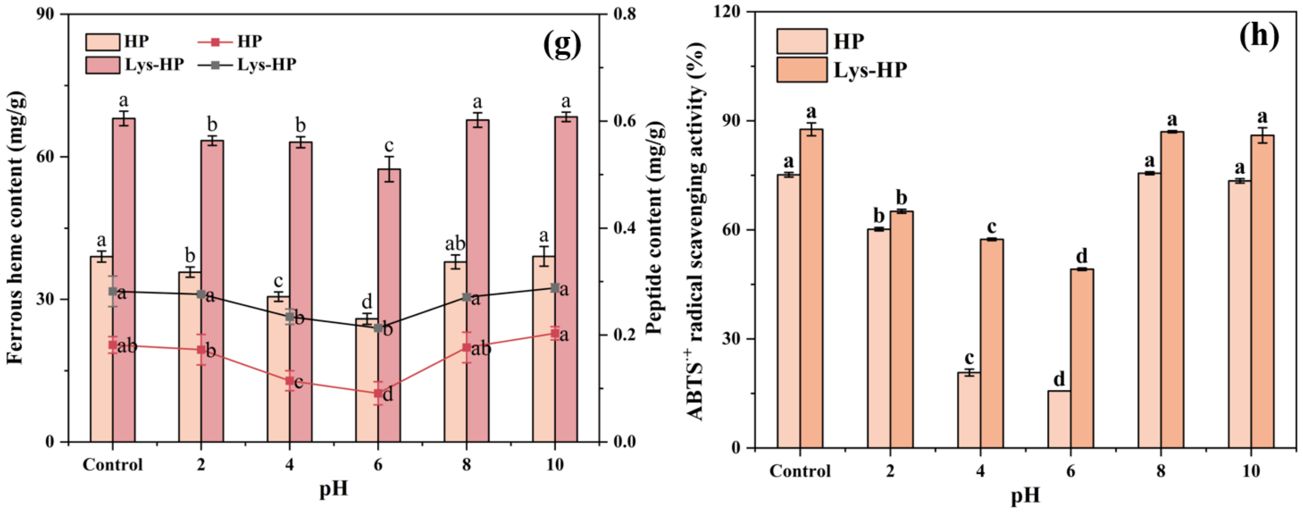


**Figure. S1.** The effects of **(a, b)** time; **(c, d)** metal ions; **(e, f)** temperature; and **(g, h)** pH on the ferrous heme content, peptide content, and the ABTS$^{+}$ radical scavenging activity of heme-peptides. Results are presented as means ± SD (n = 3). Lowercase letters indicate significant differences among columns of the same color, $P < 0.05$.
